# The Coreidae of Honduras (Hemiptera: Coreidae)

**DOI:** 10.3897/BDJ.5.e13067

**Published:** 2017-06-05

**Authors:** Carlos A Linares, Jesus Orozco

**Affiliations:** 1 Insect Collection, Zamorano University, Zamorano, Honduras

**Keywords:** Taxonomy, diversity, agriculture, pest, Central America.

## Abstract

**Background:**

Coreidae bugs are mostly sap-sucking insects feeding on a variety of plants. Despite their abundance and economic importance in Honduras there is little information on the species, their distribution and affected crops. Since knowledge of pest species allows for better management of crops, we aimed to document the diversity of this economically important group. Specimens from four entomological collections in Honduras were studied and an exhaustive search of all available literature was conducted.

**New information:**

A total of 2,036 insects were examined. The fauna of Honduran coreids is now composed of 68 species. Nineteen species are recorded for the country for the first time and 17 species were found only in literature. Little is known about the biology and economic importance of most of the species.

## Introduction

Bugs of the Coreidae family are primarily phytophagous insects that feed on plants sucking sap from branches, leaves, flowers and fruits. Many coreids are known pests of ornamentals and crops that can, at times, cause serious damage or even total loss (Henry 2009). Due to their feeding habits, they can cause malformations, rotting, discoloration, and also abortion of the fruit and poor formation of the seed (Mitchell 2000).

Honduras’ economy is largely depending on agriculture. Given that pest control depends heavily on proper species identification and management, knowledge of the species becomes paramount for the economy. Despite this, little information on insects from the country is available.

Evans and Halbert (2007) found 26 new aphids in Honduras out of the 46 species known for the country. In Coleoptera, Turnbow et al. (2003b), found that of the 626 species of Honduran cerambycids, 364 were new country records. Similarly, Turnbow et al. (2003a), found 78 bruchids new to the country of the 148 known species. We estimate that at least half of the species of insects in Honduras are known unknowns: species already described that are not recorded for the country. This, together with the relative lack of in-country taxonomists poses a difficulty for, among other things, crop management. It is impossible to know the pest status of an unknown insect.

This work is an effort to provide comprehensive information on the coreid species from Honduras, including their geographic distribution by department and the plants they are known to feed on.

## Materials and methods

Specimens from the following entomological collections in Honduras were examined:

- Colección Entomológica del Centro Universitario Regional del Litoral Atlántico, Ceiba (CURLA).
- Colección Entomológica de la Escuela Nacional de Ciencias Forestales, Siguatepeque (CEEF).
- Museo Entomológico de la Universidad Nacional Autónoma de Honduras, Tegucigalpa (UNAH).
- Zamorano Insect Collection, Zamorano University, Zamorano (EAPZ).

The material was identified by comparison with a reference collection at EAPZ and by using available keys. Label information containing host, date of collection, and distribution was recorded. Additional species information was gathered from the literature.

Geographic and temporal distribution of adults in Honduras as well as biological infomation for all species was obtained from label data. For the new country records the known distribution, outside Honduras, according to the available literature is included.

## Checklists

### List of Honduran Coreids

#### Acanthocephala alata

(Burmeister, 1835)

##### Distribution

Atlántida, Comayagua, Francisco Morazán, and Yoro.

##### Notes

Specimens examined: 115 (CEEF, CURLA, EAPZ, UNAH).

Temporal distribution: May‒September.

Hosts: *Phaseolus vulgaris* L. (beans) (EAPZ); *Annona muricata* L. (soursop) (Hernández and Pinzón 2015), and *Jatropha curcas* L. (Barbados nut) (Grimm and Maes 1997).

Attacks the fruits of *Annona muricata* L. causing their fall (Hernández and Pinzón 2015).

#### Acanthocephala declivis

(Say, 1832)

##### Distribution

Atlántida, Comayagua, and Francisco Morazán.

##### Notes

Specimens examined: 13 (CEEF, CURLA, EAPZ, UNAH).

Temporal distribution: May‒July.

Hosts: *Baccharis halimifolia* L. (groundsel bush), *Baccharis neglecta* Britton, and *Persea borbonia* (L.) Spreng. (redbay) (McPherson et al. 2011).

#### Acanthocephala femorata

(F., 1775)

##### Distribution

Atlántida, Comayagua, El Paraíso, Francisco Morazán, and Valle.

##### Notes

Specimens examined: 108 (CEEF, CURLA, EAPZ, UNAH).

Temporal distribution: January (Passoa 1983), May‒July, October.

Hosts: *Ipomoea batatas* (L.) Lam. (sweet potato), *Citrus sinensis* (L.) Osbeck (orange), *Cucurbita pepo* L. (pumpkin) (EAPZ); *Solanum tuberosum* L. (potato) (Passoa 1983); *Helianthus annuus* L. (sunflower), *Cirsium texanum* Buckley, *Ambrosia trifida* L. (wild hemp), *Baccharis neglecta* Britton, *Chenopodium album* L. (pigweed), *Cirsium horridulum* Michx. (yellow thistle), *Ratibida columnifera* (Nutt.) Wooton & Standl. (prairie coneflower), *Sorghum halepense* (L.) Pers. (Johnson grass), *Erigeron quercifolius* Lam., *Abelmoschus esculentus* (L.) Moench (gumbo), *Parthenium* sp., *Gossypium hirsutum* L. (cotton), *Prunus persica* (L.) Batsch (peach), *Sorghastrum nutans* (L.) Nash (McPherson et al. 2011), and *Jatropha curcas* L. (Barbados nut) (Alonso and Lezcano 2014).

*Acanthocephala femorata* is parasitized by *Trichopoda pennipes* (F.), (Diptera: Tachinidae). McPherson et al. (2011) recorded *Arilus cristatus* L. (Hemiptera: Reduviidae), *Gryon floridanum* Ashmead (Hymenoptera: Scelionidae), and *Bicyrtes quadrifasciata* Say (Hymenoptera: Crabronidae) as natural enemies.

#### Anasa bellator

(F., 1787)

##### Distribution

Unknown in Honduras (Maes and Goellner-Scheiding 1993).

##### Notes

Temporal distribution: July‒October (Maes and Goellner-Scheiding 1993).

Hosts: *Tournefortia* sp., *Zea mays* L. (corn), *Coffea arabica* L. (coffee) (Maes and Goellner-Scheiding 1993), and *Myristica fragrans* Houtt. (nutmeg) (Brailovsky 1985).

#### Anasa capaneodes

Stål, 1862

##### Distribution

Francisco Morazán.

##### Notes

Specimens examined: 5 (EAPZ).

Temporal distribution: May‒July.

Hosts: *Tillandsia bourgaei* Baker, *Tillandsia intumescens* L. B. Sm. and *Tillandsia prodigiosa* (Lem.) Baker (Maes and Goellner-Scheiding 1993).

#### Anasa linnavuorii

Brailovsky, 2016

##### Distribution

Yoro (Brailovsky 2016)

##### Notes

Temporal distribution: June (Brailovsky 2016).

Hosts: Unknown (Brailovsky 2016).

#### Anasa maculipes

Stål, 1862

##### Distribution

Olancho (Brailovsky 1985).

##### Notes

Temporal distribution: Unknown.

Hosts: *Cucurbita pepo* L. (pumpkin), *Quercus* sp., *Buddleja americana* L., and *Ageratina adenophora* (Spreng.) R.M. King & H. Rob. (crofton-weed) (Brailovsky 1985).

#### Anasa scorbutica

(F., 1775)

##### Distribution

Atlántida, Choluteca, Comayagua, El Paraíso, Francisco Morazán, La Paz, Olancho, and Yoro.

##### Notes

Specimens examined: 35 (CEEF, CURLA, EAPZ, UNAH).

Temporal distribution: May‒December.

Hosts: *Cucurbita argyrosperma* K. Koch, *Zea mays* L. (corn), *Cucurbita pepo* L. (pumpkin) (Passoa 1983); *Solanum lycopersicum* L. (tomato), *Lagenaria siceraria* (Molina) Standl. (bottle gourd) (Brailovsky 1985), and *Jatropha curcas* L. (Barbados nut) (Alonso and Lezcano 2014).

Brailovsky (1985) recorded *Ooencyrtus submetallicus* Howard (Hymenoptera: Encyrtidae), *Anastatus diversus* Gahan (Hymenoptera: Eupelmidae), and *Gryon carinatifronsuede* Say (Hymenoptera: Crabronidae) as natural enemies.

#### Anasa trilineata

Stål, 1870

##### Distribution

El Paraíso and Francisco Morazán.

##### Notes

**NEW COUNTRY RECORD**

Specimens examined: 11 (EAPZ).

Temporal distribution: May‒July.

Hosts: *Sechium edule* (Jacq.) Sw. and *Zea mays* L. (corn) (EAPZ).

Known distribution: Brazil, Colombia, Costa Rica, Ecuador, Nicaragua, Paraguay, Peru, and Venezuela (Packauskas 2010).

#### Anasa tristis

(De Geer, 1773)

##### Distribution

Francisco Morazán.

##### Notes

Specimens examined: 6 (CEEF, EAPZ, UNAH).

Temporal distribution: May‒July.

Hosts: *Cucurbita argyrosperma* K. Koch, *Cucurbita pepo* L. (pumpkin), *Citrullus vulgaris* Schrad. (watermelon), and *Cucumis melo* L. (sweet melon) (Alston and Barnhill 2008).

In the United States this species is considered as one of the important pests of pumpkin and squash. It causes necrosis on the leaves, scars on the fruits and a rapid wilting of the plant. It is parasitized by *Trichopoda pennipes* (F.) (Diptera: Tachinidae) (Alston and Barnhill 2008).

#### Anasa uhleri

Stål, 1868

##### Distribution

Cortés (Brailovsky 1985).

##### Notes

Temporal distribution: Unknown.

Hosts: *Cucurbita pepo* L. (pumpkin), *Opuntia streptacantha* Lem. and *Ageratina adenophora* (Spreng.) R.M. King & H. Rob. (Brailovsky 1985).

#### Anisoscelis affinis

Westwood, 1840

##### Distribution

Atlántida, Francisco Morazán, and Olancho.

##### Notes

Specimens examined: 40 (CEEF, CURLA, EAPZ, UNAH).

Temporal distribution: May‒November.

Hosts: *Passiflora edulis* Sims (passion fruit) (EAPZ), *Passiflora quadrangularis* L. (badea) (Lerma et al. 1986), and *Solanum betaceum* Cavanilles (tree tomato) (Lucas et al. 2010).

It is considered as one of the main pests of tree tomato in Ecuador (Lucas et al. 2010).

#### Camptischium clavipes

(F., 1803)

##### Distribution

Atlántida.

##### Notes

**NEW COUNTRY RECORD**

Specimens examined: 8 (EAPZ).

Temporal distribution: February.

Hosts: *Solanum melongena* L. (eggplant) (King and Saunders 1984).

Known distribution: Argentina, Bolivia, Brazil, Guyana, Lesser Antilles, Panama, and Uruguay (Packauskas 2010).

This species sucks sap from tender shoots and fruits on eggplant plantations. It can cause decay and deformation to fruits (King and Saunders 1984).

#### Catorhintha apicalis

(Dallas, 1852)

##### Distribution

Distribution in Honduras unknown (Packauskas 2010).

##### Notes

Temporal distribution: June‒September (Báez and Cervantes 2014).

Hosts: *Mirabilis jalapa* L., *Nolina parviflora* Kunth (Hemsl.), *Sphaeralcea* sp., and *Gossypium* sp. (Báez and Cervantes 2014).

#### Catorhintha guttula

(F., 1794)

##### Distribution

Atlántida, Choluteca, Comayagua, and La Paz.

##### Notes

Specimens examined: 1 (CURLA).

Temporal distribution: July.

Hosts: *Phaseolus vulgaris* L. (beans), *Zea mays* L. (corn) (Passoa 1983); *Mirabilis jalapa* L. (Cervantes et al. 2014); *Cirsium* sp., *Artemisia vulgaris* L., *Cucurbita pepo* L. (pumpkin), *Lyonia* sp., *Crotalaria* sp., *Glycine max* (L.) Merr. (soy), *Gossypium hirsutum* L. (cotton), *Sida* sp., *Mimosa pudica* L. *Mirabilis* sp. *Ricinus* sp., *Boerhavia* sp., *Oryza sativa* L. (rice), *Solanum lycopersicum* L. (tomato), *Theobrom*a sp., and *Waltheria americana* L. (Maes and Goellner-Scheiding 1993).

#### Catorhintha selector

Stål, 1860

##### Distribution

Distribution in Honduras unknown (Brailovsky and Garcia 1987).

##### Notes

Hosts: *Gossypium herbaceum* L. (cotton), *Boerhaavia diffusa* L., *Coffea arabica* L. (coffee), *Waltheria* sp. (Maes and Goellner-Scheiding 1993), and *Mirabilis jalapa* L. (Cervantes et al. 2014).

This species is frequently associated with Nyctaginaceae (Cervantes et al. 2014).

#### Cebrenis danieli

Brailovsky, 1995

##### Distribution

Comayagua (Brailovsky 1995).

##### Notes

Temporal distribution: Unknown.

Hosts: *Neurolaena lobata* (L.) R. Br. ex Cass., *Mikania scandens* (L.) Willd., and *Verbesina* sp. (Brailovsky 1995).

#### Cebreniscella exitiosa

(Brailovsky, 1984)

##### Distribution

Distribution in Honduras unknown (Packauskas 2010).

##### Notes

Temporal distribution: Unknown.

Hosts: Unknown.

#### Cebrenistella caltumae

Brailovsky, 2013

##### Distribution

Distribution in Honduras unknown (Brailovsky 2013).

##### Notes

Temporal distribution: Unknown.

Hosts: Unknown.

#### Chariesterus moestus

Burmeister, 1835

##### Distribution

Comayagua and Francisco Morazán.

##### Notes

Specimens examined: 11 (EAPZ).

Temporal distribution: May, June, July, October, and December.

Hosts: *Oryza sativa* L. (rice), *Asparagus officinalis* L. (asparagus) (EAPZ), and *Jatropha curcas* L. (Barbados nut) (Alonso and Lezcano 2014).

Recorded in Fabaceae (Maes and Goellner-Scheiding 1993).

#### Chelinidea tabulata

(Burmeister, 1835)

##### Distribution

Comayagua and Francisco Morazán.

##### Notes

Specimens examined: 6 (CEEF).

Temporal distribution: May and October.

Hosts: *Opuntia pilifera* F.A.C. Weber and *Opuntia imbricata* (Haw.) DC. (Brailovsky et al. 1994).

#### Cimolus vitticeps

Stål, 1862

##### Distribution

Distribution in Honduras unknown (Packauskas 2010).

##### Notes

Temporal distribution: Unknown.

Hosts: Unknown.

#### Diactor bilineatus

(F., 1803)

##### Distribution

Atlántida.

##### Notes

**NEW COUNTRY RECORD**

Number of specimens: 2 (CURLA).

Temporal distribution: July.

Hosts: *Passiflora edulis* Sims (passion fruit) (Oliveira and Frizzas 2014).

Known distribution: Brazil (Packauskas 2010).

It is considered one of the most important pests of passion fruit in Brazil (Oliveira and Frizzas 2014).

#### Holhymenia histrio

(F., 1803)

##### Distribution

Atlántida, Francisco Morazán, and Olancho.

##### Notes

**NEW COUNTRY RECORD**

Specimens examined: 9 (EAPZ)

Temporal distribution: February‒July.

Hosts: *Passiflora edulis* Sims (passion fruit) (EAPZ), *Passiflora nitida* Kunth, *Passiflora coccinea* Aublet, *Passiflora gibertii* Brown, and *Passiflora alata* Curtis (Baldin and Boiça 1999).

Known distribution: Argentina, Brazil, Colombia, French Guiana, Nicaragua, Paraguay, Suriname, and Uruguay (Packauskas 2010).

This species is considered an important pest of passion fruit in Brazil (Baldin and Boiça 1999).

#### Hypselonotus fulvus

(De Geer, 1773)

##### Distribution

Atlántida and La Paz.

##### Notes

Specimens examined: 5 (CURLA).

Temporal distribution: August‒September.

Hosts: *Zea mays* L. (corn), *Phaseolus vulgaris* L. (beans) (Passoa 1983), (Maes and Goellner-Scheiding 1993); *Ananas comosus* L. (pineapple) (Arellano et al. 2015), *Psidium guajava* L., *Persea americana* Miller (avocado), and *Gossypium hirsutum* L. (cotton) (Pires et al. 2013).

#### Hypselonotus interruptus

Hahn, 1833

##### Distribution

Atlántida, Choluteca, Comayagua, Cortés, El Paraíso, Francisco Morazán, Gracias a Dios, and Yoro.

##### Notes

Specimens examined: 102 (CEEF, CURLA, EAPZ, UNAH).

Temporal distribution: February‒July.

Hosts: *Eupatorium* sp., *Rubus adenotrichos* Schltdl., *Gossypium hirsutum* L. (cotton),

Hosts: *Litchi chinensis* Sonn., *Oryza sativa* L. (Rice) (Passoa 1983); *Ananas comosus* L. (pineapple) (Arellano et al. 2015); *Citrus limon* (L.) Osbeck (lemon) (USDA 2015); *Casearia sylvestris* Swartz, *Campomanesia xanthocarpa* (Mart.) O. Berg., *Myrciaria rivularis* (Cambess) O. Berg, *Acacia meanrsii* De Wild., *Caesalpinia pluviosa* DC., and *Cupressus macrocarpa* Hart. (cypress) (Thum and Costa 1997).

#### Hypselonotus lineatus

Stål, 1862

##### Distribution

Atlántida and Francisco Morazán.

##### Notes

Specimens examined: 9 (CURLA, EAPZ).

Temporal distribution: January, May‒July.

Hosts: *Annona reticulata* L. (custard‒apple), *Glycine max* (L.) Merr. (soy), *Sorghum halepense* (L.) Pers. (Johnson grass) (EAPZ), and *Jatropha curcas* L. (Barbados nut) (Alonso and Lezcano 2014).

#### Hypselonotus punctiventris

Stål, 1862

##### Distribution

Atlántida, Choluteca, Comayagua, Copán, Francisco Morazán, and Yoro.

##### Notes

Specimens examined: 119 (CEEF, EAPZ).

Temporal distribution: April‒July and September‒October.

Hosts: *Sesamum indicum* L. (sesame), *Sorghum halepense* (L.) Pers. (Johnson grass) (EAPZ); *Ipomoea batatas* (L.) Lam. (sweet potato), *Zea mays* L. (corn), *Coffea arabica* L. (coffee), *Citrus* sp. (Passoa 1983), and *Cirsium vulgare* (Savi) Tenore (Chordas et al. 2011).

#### Leptoglossus brevirostris

Barber, 1862

##### Distribution

Olancho.

##### Notes

**NEW COUNTRY RECORD**

Specimens examined: 1 (EAPZ).

Temporal distribution: August.

Hosts: *Zea mays* L. (corn) (EAPZ) and *Phoradendron leucarpum* (Raf.) Reveal & M. C. Johnst. (Whittaker 1984)

Known distribution: Costa Rica, Mexico, and United States (Packauskas 2010).

#### Leptoglossus cinctus

(Herrich-Schäffer, 1836)

##### Distribution

Atlántida, Comayagua, Francisco Morazán, and Yoro.

##### Notes

Specimens examined: 39 (CEEF, CURLA, EAPZ, UNAH).

Temporal distribution: October‒November.

Hosts: *Citrus* sp., *Psidium guajava* L. (guava) (EAPZ), *Cereus* sp., and *Opuntia* sp. (Maes and Goellner-Scheiding 1993).

#### Leptoglossus concolor

(Walker, 1871)

##### Distribution

Atlántida, Comayagua, Francisco Morazán, Gracias a Dios, and Yoro.

##### Notes

Specimens examined: 17 (EAPZ).

Temporal distribution: May, July, September, and December.

Hosts: *Anacardium occidentale* L. (cashew), *Bixa orellana* L., *Psidium guajava* L., and *Litchi chinensis* Sonn. (Mitchell 2000).

#### Leptoglossus crassicornis

(Dallas, 1852)

##### Distribution

Yoro.

##### Notes

**NEW COUNTRY RECORD**

Specimens examined: 3 (CURLA).

Temporal distribution: August.

Hosts: *Harrisia pomanensis* (F.A.C. Weber ex K. Schum.) Britton & Rose, *Opuntia anacantha* Speg., *Opuntia elata* Link & Otto ex Salm-Dyck, *Opuntia discolor* Britton & Rose, *Opuntia sulphurea* Gillies ex Salm-Dyck, *Opuntia paraguayensis* K. Schum., *Opuntia quimilo* K. Schum., and *Opuntia ficus-indica* (L.) Mill. (Coscarón and Pall 2015).

Known distribution: Argentina, Bolivia, Colombia, Paraguay, and Uruguay (Packauskas 2010). This is the first record for Central America of a species previously believed to be restricted to South America. Further research is needed to clarify the status of this species.

#### Leptoglossus gonagra

(F., 1775)

##### Distribution

Choluteca, Comayagua, Francisco Morazán, and Olancho.

##### Notes

Specimens examined: 40 (CEEF, EAPZ)

Temporal distribution: January, May‒July, September, and October.

Hosts: *Luffa cylindrica* (L.) M. Roem., *Passiflora edulis* Sims (Passion fruit), *Citrullus lanatus* (Thunb.) Matsum. & Nakai (watermelon) (EAPZ); *Momordica charantia* L. (bitter melon), *Citrus sinensis* (L.) Osbeck (oranges), *Punica granatum* L. (grenade), *Mangifera indica* L. (mango), *Nicotiana tabacum* L. (tobacco), *Cucumis melo* L. (sweet melon), *Citrus paradisi* Macfadyen (grapefruit) (Mitchell 2000), and *Jatropha curcas* L. (Barbados nut) (Alonso and Lezcano 2014).

#### Leptoglossus lineosus

(Stål, 1862)

##### Distribution

Atlántida and Francisco Morazán.

##### Notes

**NEW COUNTRY RECORD**

Specimens examined: 2 (EAPZ, UNAH).

Temporal distribution: March‒April.

Hosts: *Cucurbita* sp. (Mitchell 2000).

Known distribution: Mexico (Packauskas 2010).

#### Leptoglossus oppositus

(Say, 1832)

##### Distribution

Atlántida and Francisco Morazán.

##### Notes

Specimens examined: 1 (EAPZ).

Temporal distribution: May‒July.

Hosts: *Pinus* sp., *Cucumis sativus* L. (cucumber) (Mitchell 2000); *Helianthus* sp., *Cucurbita* sp., *Citrullus lanatus* (Thunb.) Matsum. & Nakai (watermelon), *Carya* sp., *Yucca* sp., *Gossypium* sp., *Morus* sp., *Psidium guajava* L., *Zea mays* L. (corn), *Prunus* sp., *Pyrus* sp., *Coffea arabica* L. (coffee), *Datura* sp., and *Solanum lycopersicum* L. (tomato) (Maes and Goellner-Scheiding 1993).

Maes and Goellner-Scheiding (1993) recorded *Bicyrtes quadrifasciatus* Lepeletier (Hymenoptera: Crabronidae) as a natural enemy in Nicaragua. Adults are known to be parasitized by *Trichopoda pennipes* (F.) (Diptera: Tachinidae) (Mitchell 2000).

#### Leptoglossus zonatus

(Dallas, 1852)

##### Distribution

Atlántida, Choluteca, Comayagua, Copán, Cortés, El Paraíso, Francisco Morazán, Gracias a Dios, Islas de la Bahía, Lempira, Olancho, and Yoro.

##### Notes

Specimens examined: 195 (CEEF, CURLA, EAPZ, UNAH).

Temporal distribution: Year long.

Hosts: *Luffa cylindrica* Miller, *Passiflora edulis* Sims (passion fruit), *Asparagus officinalis* L. (asparagus), *Citrus sinensis* (L.) Osbeck (orange), *Solanum lycopersicum* L. (tomato), *Zea mays* L. (corn), *Punica granatum* L. (grenada), *Anacardium occidentale* L. (cashew), *Psidium guajava* L., *Oryza sativa* L. (rice), *Solanum tuberosum* L. (potato) (EAPZ); *Cucurbita* sp., *Triadica sebifera* (L.) Small (Chinese tallow), *Sorghum* sp. (sorghum), *Schizocarpum reflexum* Rose, *Chilopsis linearis* (Cav.) Sweet, *Jatropha curcas* L. (Barbados nut), *Actinocheita filicina* (DC.) F. A. Barkley (Mitchell 2000); *Helianthus* sp., *Crescentia* sp., *Hylocereus* sp., *Schizocarpum* sp., *Cucumis* sp., *Phaseolus* sp., *Persea* sp., *Gossypium* sp., *Azadirachta indica* A. Juss., *Musa* sp., *Sesamum indicum* L. (sesame), and *Coffea arabica* L. (coffee) (Maes and Goellner-Scheiding 1993).

*Leptoglossus zonatus* is one of the most important and abundant pest species of coreids in Honduras. This species is parasitized by wasps of the genera *Geyon, Ooencyrtus, Anastatus* and *Neorileya*. *Beauveria bassiana* and *Metarhizium anisopliae* have been used successfully as a control (Mitchell 2000).

#### Leptoscelis quadrisignatus

(Distant, 1881)

##### Distribution

Distribution in Honduras unknown (Packauskas 2010).

##### Notes

Temporal distribution: Unknown.

Hosts: Unknown.

#### Lycambes andicola

(Breddin, 1903)

##### Distribution

Atlántida, Comayagua, and Francisco Morazán.

##### Notes

**NEW COUNTRY RECORD**

Specimens examined: 4 (CEEF, CURLA, EAPZ, UNAH).

Temporal distribution: July‒September.

Known distribution: Bolivia (Packauskas 2010).

#### Machtima mexicana

Stål, 1870

##### Distribution

Francisco Morazán.

##### Notes

**NEW COUNTRY RECORD**

Specimens examined: 7 (EAPZ, UNAH).

Temporal distribution: May, June, and August.

Known distribution: Mexico and Panama (Packauskas 2010).

#### Madura perfida

Stål, 1862

##### Distribution

Distribution in Honduras unknown (Packauskas 2010).

##### Notes

Temporal distribution: Unknown.

Hosts: *Zea mays* L. (corn) (Gibson and Carrillo 1959).

#### Melucha phyllocnemis

(Burmeister, 1835)

##### Distribution

Francisco Morazán.

##### Notes

**NEW COUNTRY RECORD**

Specimens examined: 1 (UNAH).

Temporal distribution: July.

Hosts: *Neurolaena lobata* (L.) R.Br. ex Cass., *Mikania scandens* (L.) Willd., and *Verbesina* sp. (Barcellos et al. 2008).

Known distribution: Bolivia, Brazil, Mexico, Colombia, and Paraguay (Packauskas 2010).

#### Melucha quadrivittis

Stål, 1862

##### Distribution

Copán, Francisco Morazán, and Olancho.

##### Notes

Specimens examined: 3 (UNAH).

Temporal distribution: May, September, and November.

#### Mozena lineolata

(Herrich-Schäffer, 1842)

##### Distribution

Atlántida, Francisco Morazán, and Olancho (Brailovsky and Barrera 2014).

##### Notes

Specimens examined: 128 (CEEF, CURLA, EAPZ, UNAH).

Temporal distribution: May‒October.

Hosts: *Prosopis* sp. and *Atriplex* sp.

#### Mozena lunata

(Burmeister, 1835)

##### Distribution

Atlántida, Comayagua, Francisco Morazán, and Yoro.

##### Notes

**NEW COUNTRY RECORD**​​​​​​​

Specimens examined: 152 (CEEF, CURLA, EAPZ, UNAH).

Temporal distribution: May‒November.

Hosts: *Malus domestica* Borkh., *Cassia* sp. (EAPZ); *Acacia farnesiana* L. Willd. (Ward et al. 1977), *Acacia amentacea* DC., and *Prosopis glandulosa* Torr. (Brailovsky et al. 1995).

Known distribution: Costa Rica, Guatemala, Mexico, Nicaragua, and United States (Packauskas 2010).

#### Mozena lurida

(Dallas, 1852)

##### Distribution

Distribution in Honduras unknown (Packauskas 2010).

##### Notes

Temporal distribution: Unknown.

Hosts: Unknown.

#### Mozena lutea

(Herrich-Schaeffer, 1840)

##### Distribution

Distribution in Honduras unknown (Packauskas 2010).

##### Notes

Temporal distribution: Unknown.

Hosts: Unknown.

#### Nematopus lepidus

Stål, 1862

##### Distribution

Atlántida, Olancho, and Yoro.

##### Notes

**NEW COUNTRY RECORD**

Specimens examined: 11 (EAPZ).

Temporal distribution: March‒August.

Hosts: *Persea americana* Miller (avocado) (EAPZ).

Known distribution: Mexico and Panama (Packauskas 2010).

#### Pachylis nervosus

Dallas, 1852

##### Distribution

Atlántida, Comayagua, El Paraíso, Francisco Morazán, Islas de la Bahía, Olancho, and Yoro.

##### Notes

Specimens examined: 255 (CEEF, CURLA, EAPZ, UNAH).

Temporal distribution: Year long.

Hosts: *Mimosa* sp. and *Prosopis laevigata* (Willd.) M. C. Johnst. (Brailovsky 1995).

#### Paryphes anceps

Horvath, 1913

##### Distribution

Cortés (Horvath 1913)

##### Notes

Temporal distribution: Unknown.

Hosts: Unknown.

#### Paryphes flavocinctus

Stål, 1860

##### Distribution

Atlántida, Francisco Morazán, and Yoro.

##### Notes

Specimens examined: 8 (CURLA, EAPZ)

Temporal distribution: April‒July.

Hosts: *Citrullus lanatus* (Thunb.) Matsum. & Nakai (watermelon) and *Gurania* sp. (Gilbert 1991).

#### Phthia lunata

(F., 1787)

##### Distribution

Atlántida, Comayagua, and Cortés.

##### Notes

**NEW COUNTRY RECORD**

Specimens examined: 5 (EAPZ).

Temporal distribution: June, August, and September.

Hosts: *Capsicum annuum* L. (sweet pepper) (EAPZ); *Citrullus lanatus* (Thunb.) Matsum. & Nakai (watermelon), and *Cucurbita* sp. (Maes and Goellner-Scheiding 1993).

Known distribution: Argentina, Brazil, Colombia, Costa Rica, Cuba, Mexico, Panama, Puerto Rico, and Suriname (Packauskas 2010).

This species is reported as a vector of *Phytomonas* sp. (Godoi et al. 2002).

#### Peranthus longicornis

(Dallas, 1852)

##### Distribution

Atlántida.

##### Notes

**NEW COUNTRY RECORD**

Specimens examined: 1 (EAPZ).

Temporal distribution: April.

Known distribution: Brazil (Packauskas 2010).

#### Phthiacnemia picta

(Drury, 1773)

##### Distribution

Atlántida, Comayagua, Choluteca, El Paraíso, and Francisco Morazán.

##### Notes

Specimens examined: 38 (CEEF, CURLA, EAPZ, UNAH).

Temporal distribution: May‒July, August, and September.

Hosts: *Ipomoea batatas* (L.) Lam. (sweet potato), *Solanum tuberosum* L. (potato), *Solanum lycopersicum* L. (tomato) (EAPZ); *Pisum* sp., *Phaseolus vulgaris* L. (beans), *Trifolium* sp., *Vicia* sp., *Vigna* sp., *Stellaria* sp., *Citrullus lanatus* (Thunb.) Matsum. & Nakai (watermelon), *Cucurbita pepo* L. (pumpkin), *Cucumis sativus* L. (cucumber), *Momordica* sp., *Passiflora edulis* Sims (passion fruit), *Punica granatum* L. (grenade), *Sorghum* sp., *Zea mays* L. (corn), *Oryza sativa* L. (rice), *Coffea arabica* L. (coffee), *Sesamum indicum* L. (sesame), *Helianthus annuus* L. (sunflower), and *Nicotiana tabacum* L. (tobacco) (Maes and Goellner-Scheiding 1993).

This species is considered an important pests of tomato in Brazil (Da Silva et al. 2003).

#### Piezogaster auriculatus

(Stål, 1862)

##### Distribution

Atlántida, Comayagua, Francisco Morazán, and Yoro.

##### Notes

Specimens examined: 54 (CEEF, CURLA, EAPZ, UNAH).

Temporal distribution: May‒October.

Hosts: *Citrus sinensis* (L.) Osbeck (oranges) and *Pachyrhizus* sp. (Dealy 2000).

#### Plapigus circumcinctus

Stål, 1860

##### Distribution

Atlántida, Comayagua, Francisco Morazán, and Yoro.

##### Notes

Specimens examined: 66 (CEEF, CURLA, EAPZ, UNAH).

Temporal distribution: May‒July and October‒December.

Hosts: *Coffea arabica* L. (coffee) (Maes and Goellner-Scheiding 1993).

#### Possaniella oblata

Brailovsky, 1999

##### Distribution

Atlántida.

##### Notes

**NEW COUNTRY RECORD**

Specimens examined: 1 (CURLA).

Temporal distribution: May.

Known distribution: Brazil (Packauskas 2010).

#### Romoniella perfecta

Brailovsky & Barrera, 2001

##### Distribution

Atlántida, Comayagua, Olancho, and Yoro.

##### Notes

Specimens examined: 9 (CEEF, EAPZ, CURLA).

Temporal distribution: February, October, and November.

#### Sagotylus confluens

(Say, 1832)

##### Distribution

Atlántida, Comayagua, Choluteca, Cortés, El Paraíso, Francisco Morazán, Islas de la Bahía, Olancho, and Yoro.

##### Notes

Specimens examined: 196 (CEEF, CURLA, EAPZ, UNAH).

Temporal distribution: Year long.

Hosts: *Oryza sativa* L. (rice), *Zea mays* L. (corn) (Passoa 1983), *Canavalia ensiformis* (L.) DC. (EAPZ); *Ricinus communis* L. (Valdés-Rodríguez et al. 2015), and *Senna obtusifolia* L. H .S. Irwin & Barneby (Palmer and Pullen 2000).

This species is considered one of the main pests of fig trees in Mexico feeding on terminal shoots and new leaves. Due to its large size, the latex loss is high and its excreta favor the establishment of fungi on the terminal buds causing them to dry (Valdés-Rodríguez et al. 2015).

#### Salamancaniella alternata

(Dallas, 1852)

##### Distribution

Atlántida and Comayagua.

##### Notes

Specimens examined: 7 (CURLA, EAPZ).

Temporal distribution: May, July, August‒November.

Hosts: *Jatropha curcas* L. (Barbados nut) (Alonso and Lezcano 2014).

#### Savius ​​jurgiosus

(Stål, 1862)

##### Distribution

Francisco Morazán.

##### Notes

**NEW COUNTRY RECORD**

Specimens examined: 2 (EAPZ).

Temporal distribution: October.

Hosts: *Baltimora* sp., *Buddleja sessiliflora* Kunth, *Prosopis* sp., and *Ziziphus* sp.

Known distribution: Costa Rica, Guatemala, Mexico, and United States (Packauskas 2010).

#### Sephina limbata

Stål, 1862)

##### Distribution

Atlántida and Comayagua.

##### Notes

Specimens examined: 13 (CEEF, CURLA)

Temporal distribution: January–April.

Hosts: Unknown.

#### Serranoniella amblysa

Brailovsky & Barrera, 2001

##### Distribution

Comayagua and Yoro.

##### Notes

**NEW COUNTRY RECORD**

Specimens examined: 22 (CEEF).

Temporal distribution: June, September‒December.

Known distribution: Brazil (Packauskas 2010).

#### Spartocera fusca

(Thunberg, 1783)

##### Distribution

Comayagua and Yoro.

##### Notes

Specimens examined: 71 (CEEF, CURLA, EAPZ, UNAH).

Temporal distribution: Year long.

Hosts: *Ipomoea batatas* (L.) Lam. (sweet potato), *Gossypium* sp. (cotton), *Solanum lycopersicum* L. (tomato), *Solanum americanum* Miller, *Physalis peruviana* L. (Maes and Goellner-Scheiding 1993); *Solanum tuberosum* L. (potato) (Passoa 1983), *Solanum nigrum* L. (Mitchell 2000), and *Capsicum annum* L. (Mitchell 2000).

Notes: *Sarcophaga sternodontis* Towns (Diptera: Tachinidae) is a known parasite of *S. fusca* in Nicaragua (Maes and Goellner-Scheiding 1993).

#### Staluptus marginalis

(Burmeister, 1835)

##### Distribution

Comayagua, Francisco Morazán, and Yoro.

##### Notes

**NEW COUNTRY RECORD**

Specimens examined: 39 (CEEF, EAPZ).

Temporal distribution: May, July, September, and October.

Hosts: *Sorghum halepense* (L.) Pers. (Johnson grass) (EAPZ); and *Cajanus cajan* (L.) Millsp. (Maes and Goellner-Scheiding 1993).

Known distribution: Guatemala and Mexico (Packauskas 2010).

#### Thasopsis formidabilis

(Distant, 1893)

##### Distribution

Atlántida, Comayagua, Francisco Morazán, and Santa Bárbara

##### Notes

**NEW COUNTRY RECORD**

Specimens examined: 13 (CURLA, EAPZ).

Temporal distribution: February, May, and September.

Known distribution: Costa Rica and Panama (Packauskas 2010).

#### Thasus acutangulus

(Stål, 1859)

##### Distribution

Atlántida and Yoro.

##### Notes

Specimens examined: 2 (CURLA, EAPZ).

Temporal distribution: December.

Hosts: *Prosopis velutina* Wooton (Torre-Bueno 1945), and *Pithecellobium* sp. (EAPZ).

#### Zicca rubricator

(F., 1803)

##### Distribution

Unknown (Brailovsky and Cadena 1992).

##### Notes

Temporal distribution: Unknown.

Hosts: *Schaueria calycobractea* Hilsenbeck & D. L. Marshall, *Chamissoa altissima* (Jacq.) Kunth and *Phytolacca rivinoides* Kunth & C. D. Bouché (Brailovsky and Cadena 1992).

#### Zicca taeniola

(Dallas, 1852)

##### Distribution

Atlántida, El Paraíso, Francisco Morazán, and Olancho.

##### Notes

Specimens examined: 32 (CURLA, EAPZ).

Temporal distribution: December.

Hosts: *Glycine max* (L.) Merr. (soy), *Solanum tuberosum* L. (potato), *Zea mays* L. (corn), *Sechium edule* (Jacq.) Sw., *Daucus carota* L. (carrot) (EAPZ), and *Capsicum annuum* L. (Maes and Goellner-Scheiding 1993).

## Discussion

A total of 2,036 specimens were examined. 68 species are now known from Honduras with 19 being new country records (Table 1). Seventeen species were only found recorded in literature.

Many of the species found in Honduras are known only from one department. We suspect this is not a real reflection of the species’ distribution but collecting bias. For nine of the species there is no information on where in Honduras they occur.

## Supplementary Material

XML Treatment for Acanthocephala alata

XML Treatment for Acanthocephala declivis

XML Treatment for Acanthocephala femorata

XML Treatment for Anasa bellator

XML Treatment for Anasa capaneodes

XML Treatment for Anasa linnavuorii

XML Treatment for Anasa maculipes

XML Treatment for Anasa scorbutica

XML Treatment for Anasa trilineata

XML Treatment for Anasa tristis

XML Treatment for Anasa uhleri

XML Treatment for Anisoscelis affinis

XML Treatment for Camptischium clavipes

XML Treatment for Catorhintha apicalis

XML Treatment for Catorhintha guttula

XML Treatment for Catorhintha selector

XML Treatment for Cebrenis danieli

XML Treatment for Cebreniscella exitiosa

XML Treatment for Cebrenistella caltumae

XML Treatment for Chariesterus moestus

XML Treatment for Chelinidea tabulata

XML Treatment for Cimolus vitticeps

XML Treatment for Diactor bilineatus

XML Treatment for Holhymenia histrio

XML Treatment for Hypselonotus fulvus

XML Treatment for Hypselonotus interruptus

XML Treatment for Hypselonotus lineatus

XML Treatment for Hypselonotus punctiventris

XML Treatment for Leptoglossus brevirostris

XML Treatment for Leptoglossus cinctus

XML Treatment for Leptoglossus concolor

XML Treatment for Leptoglossus crassicornis

XML Treatment for Leptoglossus gonagra

XML Treatment for Leptoglossus lineosus

XML Treatment for Leptoglossus oppositus

XML Treatment for Leptoglossus zonatus

XML Treatment for Leptoscelis quadrisignatus

XML Treatment for Lycambes andicola

XML Treatment for Machtima mexicana

XML Treatment for Madura perfida

XML Treatment for Melucha phyllocnemis

XML Treatment for Melucha quadrivittis

XML Treatment for Mozena lineolata

XML Treatment for Mozena lunata

XML Treatment for Mozena lurida

XML Treatment for Mozena lutea

XML Treatment for Nematopus lepidus

XML Treatment for Pachylis nervosus

XML Treatment for Paryphes anceps

XML Treatment for Paryphes flavocinctus

XML Treatment for Phthia lunata

XML Treatment for Peranthus longicornis

XML Treatment for Phthiacnemia picta

XML Treatment for Piezogaster auriculatus

XML Treatment for Plapigus circumcinctus

XML Treatment for Possaniella oblata

XML Treatment for Romoniella perfecta

XML Treatment for Sagotylus confluens

XML Treatment for Salamancaniella alternata

XML Treatment for Savius ​​jurgiosus

XML Treatment for Sephina limbata

XML Treatment for Serranoniella amblysa

XML Treatment for Spartocera fusca

XML Treatment for Staluptus marginalis

XML Treatment for Thasopsis formidabilis

XML Treatment for Thasus acutangulus

XML Treatment for Zicca rubricator

XML Treatment for Zicca taeniola

## Figures and Tables

**Table 1.** New country records of Coreidae for Honduras

**Species**	**Specimens examined**	**Collection**
*Anasa trilineata* Stål	11	EAPZ
*Camptischium clavipes* (F.)	8	EAPZ
*Diactor bilineatus* (F.)	2	CURLA
*Holhymenia histrio* (F.)	9	EAPZ
*Leptoglossus brevirostris* Barber	1	EAPZ
*Leptoglossus lineosus* (Stål)	2	EAPZ, UNAH
*Leptoglossus crassicornis* (Dallas)	3	CURLA
*Lycambes andicola* Breddin	4	All collections
*Machtima mexicana* Stål	7	EAPZ y UNAH
*Melucha phyllocnemis* (Burmeister)	1	UNAH
*Mozena lunata* (Burmeister)	152	All collections
*Nematopus lepidus* Stål	11	EAPZ
*Peranthus longicornis* (Dallas)	1	EAPZ
*Phthia lunata* (F.)	5	EAPZ
*Possaniella oblata* Brailovsky	1	CURLA
*Savius jurgiosus* (Stål)	2	EAPZ
*Serranoniella amblysa* Brailovsky & Barrera	22	CEEF
*Staluptus marginalis* (Burmeister)	39	EAPZ, CEEF
*Thasopsis formidabilis* (Distant)	13	EAPZ, CURLA
